# Cognitive and Behavioral Contributions to Depression Severity, Quality of Life, and Functioning Among People Living With HIV in South Africa

**DOI:** 10.1016/j.beth.2022.07.003

**Published:** 2022-07-16

**Authors:** Lena S. Andersen, Amelia M. Stanton, Jessica F. Magidson, John A. Joska, Conall O’Cleirigh, Jasper S. Lee, Ashraf Kagee, Jade A. Witten, Steven A. Safren

**Affiliations:** University of Copenhagen; Massachusetts General Hospital and Harvard Medical School; University of Maryland; University of Copenhagen; Massachusetts General Hospital and Harvard Medical School; University of Miami; Stellenbosch University; Bangor University; University of Miami

**Keywords:** depression, HIV, behavioral activation, rumination, South Africa

## Abstract

Cognitive-behavioral treatments for depression typically address both behavioral (e.g., activation) and cognitive (e.g., rumination) components, and consequently improve quality of life (QOL) and function in high-resource settings. However, little is known about the cross-cultural applicability and relative contribution of these components to depression symptom severity, QOL, and functional impairment in South Africa and other resource-limited global settings with high HIV prevalence rates.

Persons with HIV (*N* = 274) from a peri-urban community outside Cape Town, South Africa, were administered multiple measures of depression (Hamilton Depression Scale, Centre for Epidemiological Studies Depression Scale, South African Depression Scale), cognitive and behavioral components related to depression (Ruminative Response Scale, Behavioral Activation for Depression Scale), and measures of QOL and functioning (Sheehan Disability Scale, Quality of Life Enjoyment and Satisfaction Scale—Short Form). Multiple linear regression models were fit to assess the relative contribution of behavioral and cognitive components to depression severity, QOL, and functional impairment in this population.

Models accounting for age and sex revealed that lower levels of behavioral activation (BA) were significantly associated with all measures of depression, as well as with QOL and functional impairment (all *ps* < .01). Rumination was associated with all measures of depression (all *ps* < .01), but not with QOL or functional impairment.

The consistent and unique association of BA with depression, QOL, and functional impairment bolsters its importance as a treatment target for this population.

South africa has the greatest number of people living with HIV (PWH) in the world. Rates of depression among PWH in South Africa are particularly high compared to the general population (Freeman et al., 2008). Depression can negatively impact on health behaviors and outcomes among PWH, including adherence to treatment (Uthman et al., 2014), disease progression, and, at worst, mortality (Leserman et al., 1999). Unfortunately, a mental health treatment gap exists in South Africa, with an estimated one in four in need of mental care receiving appropriate treatment (Sheehan et al., 1998). Given the prevalence and public health significance of depression among PWH in South Africa, there is a need to better understand the disorder in order to guide development of the most effective and parsimonious treatments. This approach is imperative in a resource-constrained setting with limited health care providers to serve as interventionists (Lund et al., 2009).

In the past decade, there has been a growth in the number of trials of psychological treatments for depression among PWH in low- and middle-income countries (LMICs; Chibanda et al., 2015; Spies et al., 2013), including in South Africa (Fairall et al., 2018; Joska et al., 2020; Myers et al., 2018), to address the mental health treatment gap (Demyttenaere et al., 2004). Consistent with global mental health research, these treatments are largely adapted from evidence-based treatments from high-income countries and have been culturally adapted and task shifted for delivery by nonspecialist providers, such as nurses and lay counselors (Singla et al., 2017). These treatments mostly consist of a variety of cognitive-behavioral treatments or strategies, such as motivational interviewing, problem-solving therapy, behavioral activation (BA) therapy, and relaxation training. Some of these trials have reported promising results (Andersen et al., 2018; Safren et al., 2021; Spies et al., 2013), which could have favorable implications for implementation in public health care in South Africa. What is not yet known, and what has surprisingly not been explored in this context, is broadly whether the explanatory models of depression that these evidence-based treatments from high-income countries are based on are cross-culturally applicable, and specifically whether cognitive and behavioral constructs are associated with change in depression and other key outcomes, such as functioning and quality of life (QOL).

In high-income countries, specific cognitive and behavioral components have been identified as significant contributors to depression, notably rumination and BA-related processes (Dimidjian et al., 2011; Nolen-Hoeksema, 1991). Symptoms of depression can be categorized, generally, into cognitive symptoms (e.g., negative thoughts and beliefs, including guilt and worthlessness leading to sadness, difficulty concentrating), behavioral symptoms (e.g., loss of interest and pleasure, withdrawal), and physiological symptoms (e.g., sleep impairment, loss of energy, appetite changes). According to the ruminative response styles theory, rumination (i.e., the cognitive process of focusing on one’s depressive symptoms and the causes and implications of one’s distress without taking action) dictates the severity and duration of depressive symptoms (Morrow & Nolen-Hoeksema, 1990; Nolen-Hoeksema, 1991). In contrast, behavioral theories of depression posit that depression is developed and maintained through reductions in one’s obtainment of positive reinforcement from their environment. This may be a result of (a) a decrease in response-contingent positive reinforcement that negatively impacts on mood; and (b) behavioral avoidance, which contributes to engaging in behaviors that could provide the positive reinforcement necessary to maintain normal mood (Dimidjian et al., 2011). Both the constructs of rumination and BA are thought to contribute to the onset, severity, and duration of depression, and several cognitive-behavioral theories, largely developed and tested in high-income countries, have integrated the focus on both cognitive and behavioral components, such as rumination and BA.

In the middle-income country of South Africa, the constructs of rumination and BA seem to exist cross-culturally. In a qualitative study with PWH with clinical depression in South Africa, participants expressed local idioms of distress that seemed consistent with these constructs when describing their depression (Andersen et al., 2015). Descriptions of “thinking, thinking, thinking” and “thinking too much” seemed consistent with rumination and descriptions of “not wanting to be around people” and “wanting to be alone” seemed consistent with behavioral avoidance and withdrawal (Andersen et al., 2015). Yet little is known of the contribution of these constructs to depression cross-culturally and in PWH in South Africa specifically. Although there is a growing literature on cross-cultural similarities and differences in the experience and expression of distress (Kaiser et al., 2015; Kaiser & Weaver, 2019), limited research has examined the role cognitive-behavioral mechanisms play in the development and maintenance of depression in individuals in LMICs and in PWH in South Africa specifically. The aim of the current study was to assess whether the constructs of rumination (cognitive) and BA (behavioral) are applicable in this context and whether these constructs impact on depression severity and health-related QOL and functioning among PWH in South Africa. Understanding the contribution of these constructs to the maintenance of depression could provide valuable insight into possible mechanisms that could be targeted in treatments for this population in this context.

## Method

### PARTICIPANTS

The current study utilized baseline data from the larger Ziphamandla trial, a randomized controlled trial of the effectiveness of nurse-administered cognitive-behavioral therapy for adherence and depression (CBT-AD) among PWH with suboptimal adherence to antiretroviral therapy (ART) in South Africa (Safren et al., 2021). Participants in the current study were adults who had failed first-line ART, spoke English or isiXhosa, and met diagnostic criteria for major depressive disorder. Participants were recruited from six primary infectious disease clinics in Khayelitsha, a peri-urban area located east of Cape Town (see Joska et al., 2020, for details on design).

### PROCEDURE

As part of the baseline assessment for the parent trial, mental health nurses and trained research assistants administered a number of scales in participants’ preferred language. Data were entered into REDCap on tablets. REDCap is a secure, web-based data management platform (Harris et al., 2020). Ethics approval was granted by the University of Cape Town’s Faculty of Health Sciences Human Research Ethics Committee and the University of Miami’s Institutional Review Board. Clinic access was granted by the City of Cape Town’s Health Department.

### DEPRESSION MEASURES

#### Hamilton Depression Scale (HAM-D)

The HAM-D (Williams, 2001) is a clinician-administered measure used to assess depression. The 17 items evaluate a range of depressive symptoms experienced in the past week. Scores range from 0 to 52 with higher scores indicating greater severity. The HAM-D has been used globally in psychiatric research, including in several antidepressant medication trials in South Africa (Gagiano et al., 1994; Kennedy & Emsley, 2006). It has demonstrated strong psychometric reliability (α = .87). In the current study, the HAM-D was administered by two trained psychiatric nurses. Recordings of the HAM-D administration were reviewed by a psychiatrist, with certified HAM-D training, while providing ongoing supervision to the psychiatric nurses.

#### Center for Epidemiological Studies Depression Scale (CES-D)

The CES-D is a self-report scale used to measure depressive symptoms. The 20 items evaluate a range of depressive symptoms experienced in the past week on a 4-point Likert scale. There are four positive items on the scale that are reverse coded. Scores range from 0 to 60 with higher scores indicating greater severity. The CES-D has been used extensively in depression research globally (Tsai, 2014) and has been found to be reliable (α = .95) for use among PWH in South Africa (Kagee et al., 2020).

#### South African Depression Scale (SADS)

The SADS (Andersen et al., 2020) is a locally developed scale to measure depression in PWH in South Africa. The 16 items evaluate a range of local and traditional idioms of distress used to describe depression, such as “thinking too much,” “not wanting to be around people,” “sadness,” “carrying a lot of weight on my shoulders,” and “having pain inmyheart.”Symptoms are scored on a 4-point Likert scale, indicating how many days in the past week they were experienced (0, 1–2, 3–4, 5+). Scores range from 0 to 48 with higher scores indicating greater severity. A final, open-ended question at the end asks when the symptoms first started. The internal reliability (α = .96) and construct validity of the SADS have been established in a previous study (Andersen et al., 2020). In the current study, the SADS was administered by a trained research assistant. Notably, the SADS was added to the assessment battery after the start of the trial—therefore, fewer participants completed the SADS (*n* = 199) relative to the other depression measures (*n* = 274). We chose to include the SADS because it is highly culturally relevant, as it was developed specifically to assess depression among PWH in the South African context.

### COGNITIVE AND BEHAVIORAL MEASURES

#### Behavioral Activation Depression Scale (BADS)

The BADS (Fuhr et al., 2016; Kanter et al., 2007) is a self-report scale to measure changes in the behaviors hypothesized to underlie depression. It has demonstrated good reliability (α = .86) and internal consistency in a German sample (Fuhr et al., 2016). The 25 items are grouped into four subscales to evaluate the domains of activation, avoidance/rumination, work/school impairment, and social impairment. Responses are scored on a 7-point Likert scale indicating how much each item was experienced in the past week. The total score is calculated by reverse coding all items other than those from the Activation subscale and summing them. A higher total score indicates greater activation. No items are reverse coded when scoring the subscales individually. The BADS has been used extensively to measure changes in BA during and following treatment (Fuhr et al., 2016).

#### Rumination Response Scale (RRS)

The RRS (Roelofs et al., 2006) is a self-report scale to measure responses to depressed mood. The 22 items evaluate three factors: depression, brooding, and reflection. Responses are scored on a 4-point Likert scale indicating how often each item is done when feeling down, sad, or depressed. The total RRS score is a sum of all items. Scores range from 22 to 88 with higher scores indicating greater levels of rumination. The RRS has been found to be reliable (α = .87–.90) and it is a widely used scale to measure rumination (Luminet, 2004; Roelofs et al., 2006).

### QOL AND DISABILITY MEASURES

#### Quality of Life Enjoyment and Satisfaction Questionnaire—Short Form (Q-LES-Q-SF)

The 16 item Q-LES-Q-SF is a shortened version of the 93-item Q-LES-Q (Riendeau, 2018). It measures overall enjoyment and satisfaction in various domains of daily functioning, such as physical health, mood, work, household, and leisure activities, relationships, daily functioning, general activities, overall well-being, and medications. Responses are scored on a 5-point Likert scale indicating how often each item was experienced in the past week. The total Q-LES-Q-SF score is based on the sum of the initial 14 items. The last two items about medication and overall life satisfaction are considered separately. Scores range from 0 to 70 with higher scores indicating greater enjoyment and satisfaction with life. The Q-LES-Q has been found to be reliable (α = .90) and it has been used extensively in outcome research in psychiatry including in trauma and depression research among PWH in South Africa (Kanter et al., 2007).

#### Sheehan Disability Scale (SDS)

The SDS (Leon et al., 1997) is a three-item self-report scale that uses a 10-point Likert scale (0–10) to assess the extent to which work/school, social life, and home life/family responsibilities are impaired by an individual’s symptoms. The numerical ratings can be translated into a percentage and/or the three items can be summed into a single dimensional measure of global impairment that ranges from 0 to 30, with higher scores indicating greater impairment. Because the majority of participants were unemployed and did not answer SDS item 1, which pertains to perceived impairment at work/school, we calculated the SDS total score as a percentage of items endorsed (i.e., out of a possible score of either 20 or 30). The SDS has been found to be reliable (α = .89; Leon et al., 1997) and it has previously been used in studies of depression in South Africa (Andersen et al., 2018; Olley et al., 2004)

### DATA ANALYSES

Statistical analyses were conducted using the Statistical Package for the Social Sciences (SPSS) version 25 (IBM Corp., 2019). Descriptive statistics were calculated and presented as frequencies (%) or means (*SD*; see Table 1), and distributions were assessed for each continuous variable.

To examine the relative contributions of BA (assessed by the BADS) and rumination (assessed by the RRS) to depression symptom severity, three multiple regression models were fit, one for each measure of depression (the HAM-D, the CES-D, and the SADS) with both cognitive and behavioral components included in the same models. Demographic variables (age and gender) were included in the models to control for gender-based differences in depression (Kessler, 1993) including among PWH in South Africa (Olley et al., 2004). The assumptions for ordinary least squares (OLS; e.g., linearity, normality, homoscedasticity, multicollinearity) were examined prior to analysis, and an OLS approach was determined to be acceptable for these data. Due to significant content overlap and a strong correlation between the RRS and the Avoidance/Rumination subscale of the BADS (*r* = .62, *p* < .01), the Avoidance/Rumination subscale was removed, leaving the three other subscales (Activation, Work/School Impairment, and Social Impairment) to comprise the BADS total score.

Similarly, to examine the relative contributions of BA and rumination to QOL and functional impairment, two multiple regression models were fit, one for each outcome. Age and gender were again included as covariates.

## Results

The sociodemographic characteristics of the sample are presented in Table 1. The majority of participants were women (71.0%), and all women were cisgender; the sample included two transgender men (0.7%). The average age of all participants included in the analyses was 39.2 years (*SD* = 9.0). The majority of the sample spoke isiX-hosa at home (81.6%) and indicated they were exclusively attracted to the opposite sex (93.7%). Just under 20.0% of the sample reported being married, with the majority of participants (46.7%) selecting single as their relationship status. Unemployment rates were high (79.4%), and participants indicated an average monthly income of $138.20 (*SD* = 164.9). The average depression symptom severity score across the sample on the CES-D was 33.1 (*SD* = 13.5); among PWH in South Africa, scores of at least 20 are indicative of major depressive disorder (Myer et al., 2008). The average scores on the HAM-D and the SADS, the other two measures of depression that were included in the analyses, were 23.8 (*SD* = 8.0) and 31.0 (*SD* = 10.6), respectively. Scores of at least 8 on the HAM-D are indicative of major depressive disorder (Zimmerman et al., 2013). The mean total score of the BADS, including the Rumination/Avoidance subscale that was removed for the subsequent analyses, was 66.06 (*SD* = 24.63); the mean total score of the BADS without the Rumination/Avoidance subscale was 43.70 (*SD* = 18.63). Finally, the mean score of the RRS across the sample was 55.84 (*SD* = 13.06).

### BA, RUMINATION, AND DEPRESSION SEVERITY

In the model that used the CES-D as the outcome variable, BA, B = −0.41, *p* < .001, 95% CI [−0.49, −0.33], and Rumination, B = 0.23, *p* < .001, 95% CI [0.12, 0.33], were significantly associated with depression severity—that is, a 1.0 unit increase in BA was associated with a 0.41 unit decrease in depression severity and a 1.0 unit increase in rumination was associated with a 0.23 unit increase in depression severity.

When the BADS subscales (Activation, Work Impairment, and Social Impairment) were included in the model instead of the BADS total score, all were significantly associated with depression severity, Activation: B = −0.49, *p* < .001, 95% CI [−0.63, −0.35; Work Impairment: B = 0.33, *p* = .003, 95% CI [0.12, 0.55]; and Social Impairment: B = 0.38, *p* < .001, 95% CI [0.20, 0.56].

In the model that used the HAM-D as the outcome variable, both BA, B = −0.18, *p* < .001, 95% CI [−0.24, −0.13], and Rumination, B = 0.12, *p* = .005, 95% CI [0.04, 0.20], were significantly associated with depression severity; a 1.0 unit increase in BA was associated with a 0.18 unit decrease in depression severity, and a 1.0 unit increase in rumination was associated with a 0.12 unit increase in depression. The same was true for the model in which the SADS served as the outcome variable, with BA, B = −0.31, *p* < .001, 95% CI [−0.38, −0.24], and Rumination, B = 0.19, *p* = .001, 95% CI [0.08, 0.29], significantly contributing to depression severity. Specifically, a 1.0 unit increase in BA was associated with a 0.31 unit decrease in depression as measured by the SADS, whereas a 1.0 unit increase in rumination was associated with a 0.19 unit increase in depression severity.

Again, when the three BADS subscales were included in the model instead of the BADS total score, all were significantly related to depression severity as measured by the HAM-D, Activation: B = −0.18, *p* = .001, 95% CI [−0.29, –0.08]; Work Impairment: B = 0.22, *p* = .009, 95% CI [0.06, 0.38]; and Social Impairment: B = 0.15, *p* = .02, 95% CI [0.02, 0.29].

### BA, RUMINATION, AND QOL

BA was significantly associated with QOL, B = 0.004, *p* < .001, 95% CI [0.003, 0.005], whereas rumination was not. Specifically, a 1.0 unit increase in BA was associated with a 0.004 unit increase in QOL.

In the model that included the three BADS subscales instead of the BADS total score, Activation, B = 0.005, *p* < .001, 95% CI [0.005, 0.007], and Social Impairment, B = −0.003, p = .002, 95% CI [−0.005, −0.001], were significantly associated with QOL, whereas Work Impairment was not.

### BA, RUMINATION, AND FUNCTIONAL IMPAIRMENT

In the model that assessed the degree to which the mechanisms of depression were related to functional impairment, BA was significantly associated with impairment, B = −0.009, *p* = .04, 95% CI [−0.015, 0.0]—that is, each unit increase in BA was associated with a 0.009 unit decrease in functional impairment. Rumination was not significantly associated with functional impairment in this sample.

When the three BADS subscales were used in the model instead of the BADS total score, none of the subscales were individually associated with functional impairment.

## Discussion

Consistent with the literature from high-income countries, rumination and BA both significantly contributed to depression as measured by the HAM-D, CES-D, and the local measure of depression, the SADS. BA was also found to significantly contribute to QOL and level of functioning, suggesting that withdrawing from value-driven, reinforcing activities that provide a sense of pleasure and accomplishment is a central contributor to low mood, QOL, and functioning among PWH in South Africa.

The finding that BA substantially contributes to depression among PWH has important implications for treatment development in this resource-constrained setting. BA therapy, which directly targets increasing value-driven, reinforcing activities to improve mood, would be an important component of depression treatment among PWH in South Africa. Given the resource constraints of LMICs including South Africa, task-shifting mental health care delivery to nonspecialist providers is the recommended approach (Kakuma et al., 2011; World Health Organization, 2008). Because BA therapy is relatively straightforward to train in and to administer, and it targets a significant contributor to depression, it may be an ideal option for a task-shifted depression treatment in this population.

Rumination was also found to be a significant contributor to depression and “thinking too much” is often experienced by PWH suffering from depression in South Africa and other sub-Saharan countries (Andersen et al., 2015; Kidia et al., 2015). Surprisingly, rumination was not significantly associated with QOL or functional impairment in this sample. Given that rumination contributes to depression, and depression negatively impacts on QOL and functioning, it seems reasonable to expect rumination to be associated with impaired QOL and functioning. Perhaps there are culture-specific protective factors for well-being in depressed people in the South African context, given that subjective well-being is a “multifactorially determined construct” (Kuehner & Buerger, 2005). Religiosity and social support are well-established protective factors for QOL. Given that Xhosa is a collectivist culture with strong social ties (Eaton & Louw, 2000) and over 90% of the current sample indicated that they were religious, perhaps these characteristics promote well-being independent of rumination in this context. Further investigation of the relationship between rumination and QOL, and the potential role of religiosity and social support, is warranted, particularly in this population living with comorbid HIV.

With regard to the nonassociation between rumination and level of functioning, it seems reasonable in a low-income community where multiple, extended family members often rely on one another for income, child care, food provision, and so on, that the rumination severity threshold for impaired functioning would be higher than in high-income communities. Rumination contributes to depression, but it does not, on its own, seem to reduce level of functioning in this context where functioning is imperative to group survival. Alternatively, it could be an issue with the cross-cultural definition or measurement of rumination rather than a true nonassociation. The RRS was developed in a high-income country and has not yet been validated in South Africa. Further exploration is needed on the concept of rumination and its association with QOL and functioning in PWH in South Africa.

To develop and deliver the most parsimonious treatment for depression in this population, it would be interesting to explore whether BA could indirectly mitigate rumination or whether cognitive strategies that directly target rumination are required for symptom relief. The findings of a noninferiority trial by Richards et al. (2016) of task-shifted BA compared with cognitive-behavioral therapy (CBT) for depression suggest that BA alone is as effective as CBT in treating depression in the United Kingdom. This is consistent with the study by Jacobson et al. (1996) in the United States that found BA to be as effective as the full CBT treatment for depression. Whether this is the case among depressed PWH in South Africa has yet to be established. However, a recent trial by Safren and colleagues (2021) conducted among PWH in South Africa found a significant effect of a task-shifted treatment package, which included BA, in treating depression and improving adherence to ART. Further research is needed to assess changes in rumination and BA as a result of therapy modules that directly and indirectly target these components.

Limitations of the current study were the cross-sectional design and the exploration of only two constructs (one cognitive, one behavioral) hypothesized to contribute to depression rather than the assessment of a more exhaustive list of potential contributors or other examples of cognitive and behavioral components. For example, no measure of negative affective biases, posited to contribute to the onset and maintenance of depression, was included (Roiser et al., 2012). We were also unable to assess the impact of avoidance on depression since we removed the Rumination/Avoidance subscale from the BADS in our analysis due to its overlap with the RRS. Nevertheless, to our knowledge, this is the first study that examines the contribution of both cognitive and behavioral constructs to depression, QOL, and functional impairment among PWH in South Africa. A longitudinal study to determine whether depression mediates later changes in QOL and functional impairment is recommended.

The results of this study emphasize the importance of addressing rumination and BA in depression treatment for PWH in this context. It also highlights the appropriateness and potential impact of BA therapy for improving depression, QOL and functioning in this population. BA could be a particularly useful therapy module for treating depression in this resource-limited setting given the ease of training paraprofessionals in its delivery and its potential cost-effectiveness. Efforts have already been implemented to adapt BA treatment for task-shifted delivery in PWH in South Africa with other related comorbidities, including several studies by our team (Andersen et al., 2018; Safren et al., 2021; Magidson et al., 2020a, 2020b, 2021). Given that BA seems to be relevant in this context and is most strongly associated with QOL and functional impairment, it should be considered as a potential core treatment component in depression trials. Next steps include testing BA as a mechanism of treatment effectiveness.

## Figures and Tables

**Table 1:** Demographic and Clinical Characteristics of Participants (*N* = 274)

	*n*	%^a^	*M*	*SD*

Age			39.2	9.0
Gender				
Men	76	27.9		
Women	193	71.0		
Transgender men	2	0.7		
Sexual orientation				
Exclusively opposite sex partners	252	93.7		
Mostly opposite sex partners	6	2.2		
Either opposite sex or same-sex partners	2	0.7		
Mostly same-sex partners	2	0.7		
Exclusively same-sex partners	7	2.6		
Language spoken at home				
isiXhosa	222	81.6		
English	10	3.7		
Afrikaans	3	1.1		
Other	3	1.1		
Relationship status				
Single	127	46.7		
Married	53	19.5		
Living together/common law	17	6.3		
Divorced	17	6.3		
Widowed	17	6.3		
Religion^b^				
Christian	242	90.0		
Rastafarianism	1	0.4		
Muslim	0	0		
Traditional religion	33	12.3		
Monthly household income (USD)			138.2	164.9
Unemployment	216	77.9		
Engagement in HIV care				
Percent self-reported ART adherence (past 2 weeks)		69.7		
Percent of HIV appointments attended (past year)		61.7		

*Note. M* = mean; *SD* = standard deviation; USD = U.S. dollars; ART = antiretroviral therapy.

a Percentages are calculated from the total of nonmissing values.

b Total adds to more than 100% because participants could select more than one religion.
